# Multispecific drugs: the fourth wave of biopharmaceutical innovation

**DOI:** 10.1038/s41392-020-0201-3

**Published:** 2020-06-04

**Authors:** Yifei Wang, Shengyong Yang

**Affiliations:** State Key Laboratory of Biotherapy and Cancer Center, West China Hospital, Sichuan University, Chengdu, Sichuan 610041 China

**Keywords:** Drug screening

Recently, a paper by Deshaies^1^ brilliantly and systematically summarized the current hot topic, multispecific drugs, which represent the fourth revolutionary wave of biopharmaceutical innovation that is now sweeping over the biopharmaceutical industry.

**Fig. 1:**
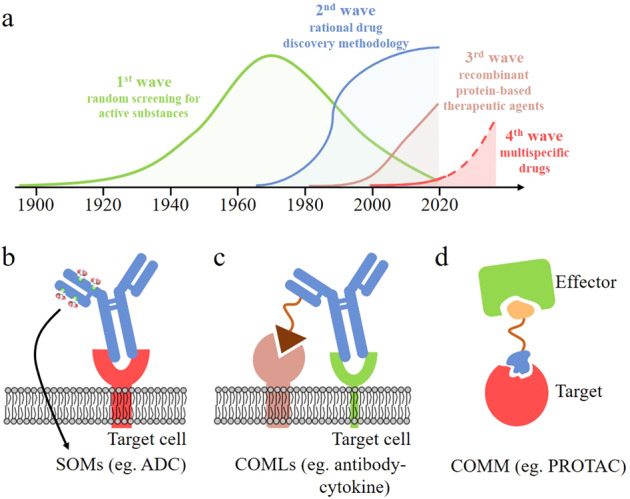
Four waves in biopharmaceutical innovation and different categories of multispecific drugs. **a** Four waves of transformative innovation in the development of drugs according to Deshaies;^1^ **b**, **c** Tetherbodies, a SOM (**b**) and a COML (**c**); **d** A matchmaker or a COMM

The modern pharmaceutical industry originated in the beginning of 20th century. The past 120 years have seen three waves of transformative innovation in the development of drugs (Fig. 1a): the first wave, namely random screening for active substances from culture broths or biological extracts, which started from the early 20th century and now seldom uses; the second wave, which is rational drug discovery methodology, beginning in the 1970s and now still dominating the drug research and development (R&D); the third wave, recombinant protein-based therapeutic agents, starting in the 1980s and still growing fast at present. We are now witnessing the coming of the fourth wave, multispecific drugs.

What are the major differences between the multispecific drugs and classical drugs? The classical drugs, including small molecule drugs and macromolecule antibodies, follow the principle of one target and one drug (1T1D). Drugs and targets are directly combined to form a clear single drug-target binding interface, which plays a role by promoting or inhibiting the function of the target. In other words, they need to occupy the active sites of target proteins to exert their bio-functions, which are typical “occupancy driven” drugs. The classical drugs usually work in the whole body and have no tissue specificity. On the contrary, multispecific drugs work through two or more entities, either limiting drug activity to a specific location, or anchoring the target close to an endogenous effector such that allowing the effector to modulate the target. Multispecific drugs must form two or more drug-target binding interfaces either sequentially or concurrently, and then their therapeutic effect can come out. Therefore, they belong to the “event driven” drugs. Multispecific drugs often have better tissue specificity.

According to Deshaies, the multispecific drugs can be roughly classified into two categories. The first category is tetherbodies, which can be further divided into two sub-classes: SOMs (sequential obligate multispecific drugs) and COMLs (concurrent obligate multispecific drugs that mediate localization). A SOM is a tetherbody that binds sequentially to two molecules in different compartments—the dock and target, and forms two interface, respectively. One interface engages the dock that enriches the drug in a particular location and another engages the target, the function of which is modified by the drug (Fig. 1b). Typical examples of SOMs include the antibody-toxin fusion moxetumomab pasudotox and antibody-drug conjugates (ADCs).^2^ Up to now, several ADCs are now on the market, including gemtuzumab ozogamicin. COMLs are very similar with SOMs, except that the dock and target are in the same compartment and must be bound simultaneously for the drug to work. An example of COMLs is an antibody-cytokine fusion.^3^ Numerous antibody-cytokine COMLs are in clinical trials, but none have been approved to enter market.

The second category of multispecific drugs is COMMs (concurrent obligate multispecific drugs that function as molecular matchmakers). COMMs or matchmakers pull two (or more) entities (the effector and target) together such that one (the effector) acts upon another (the target) (Fig. 1d). With matchmaker drugs, therapeutic modulation of the target is achieved by utilizing an endogenous biological mechanism, such as the ubiquitin-proteasome degradation system, and autophagy. Examples of COMMs include immunosuppressants and plant hormones (such as cyclosporin, auxin, brefeldin A), molecular glue (such as lenalidomide),^4^ PROTAC (proteolysis targeting chimeric) molecules,^5^ bispecific CD3 engagers (BCEs)-, and heteroduplex IgG. Some COMMs, such as cyclosporin and thalidomide, have entered market.

Compared with classical drugs, multispecific drugs have several advantages. Firstly, multispecific drugs benefit to increase efficacy and in the same time reduce toxicity. For example, the tetherbodies (SOMs and COMMs) could concentrate drugs at their relevant site of action, at which the drug exerts its therapeutic effect. This can obviously reduce on- and off-target toxicity in cells and tissues. Secondly, multispecific drugs have the potential to open the way of drug development against the vast majority of the proteome that is currently’undruggable’. For instance, “molecular glue” lenalidomide enables complex formation between CRBN and the transcription factors IKZF1 and IKZF3, which are thought as undruggable, resulting in the ubiquitylation of IKZF1 and IKZF3 and subsequent degradation by the proteasome; IKZF1 and IKZF3 are important oncogenes. Thirdly, because multispecific drugs are “event driven”, not “occupancy driven” (like classical drugs), they have a clear superiority in some aspects compared with classical drugs, such as, low dosage (like a catalyst’s amount) being sufficient to achieve a specific biological function, easy to overcome drug resistance caused by target protein mutation or overexpression, and therapeutic efficacy independent of binding affinity of drugs.

Multispecific drugs are more complex in both chemical structure and mechanism of action than classical drugs because they interact with two or more entities other than one entity in classical drugs. The R&D of multispecific drugs are therefore much more complicated than that of classical drugs. At present, there is still a lack of theoretical guidance, and many laws obtained in classic drugs, such as rule-of-5, are not applicable to multispecific drugs. Through uninterrupted efforts in the past two decades, some progress has been made, and a number of prospectively developed multispecific drugs (including Gemtuzumab ozogamicin, catumaxomab, blinatumomab, emicizumab) have been approved to enter market. Despite this progress, we have to acknowledge that the development of multispecific drugs is still in its infancy stage, and there are challenges faced in many aspects, including synthesis, quality control, structural optimization, pharmacokinetics, safety, manufacture, clinical development, and commercialization. We believe that, with the continuous exploration and technological progress, some of these challenges will be solved, and the multispecific drugs must have a bright future.
